# Evaluating Surgical Outcomes and Survival in Colon Cancer Patients Over 80 Years Old

**DOI:** 10.7759/cureus.64059

**Published:** 2024-07-08

**Authors:** Ana Sofia Cavadas, Jorge Rodrigues, Carlos Costa-Pereira, Joaquim Costa-Pereira

**Affiliations:** 1 General Surgery Department, Hospital de Braga, Braga, PRT; 2 Medical Oncology Department, Hospital de Braga, Braga, PRT

**Keywords:** surgical oncology, surgical outcomes, geriatrics, survival, colon cancer

## Abstract

Background and aims: In the context of an increasing older population, knowing the surgical outcomes of older patients is of paramount importance to define a comprehensive strategy for colon cancer treatment in these patients. This study aimed to analyze the surgical outcomes and survival of patients over 80 years old undergoing surgery for colon cancer.

Materials and methods: This is an observational retrospective longitudinal study of patients over 80 years old with colon cancer diagnosis who underwent surgery for this condition, between 2018 and 2021, in a Portuguese hospital. Demographic and clinical features were characterized. Kaplan-Meier method was used for survival analysis.

Results: Out of 90 patients in the study, 41.1% were female. The majority (56.7%) had an Eastern Cooperative Oncology Group (ECOG) performance status of 1 or 0, with a median Charlson Comorbidity Index of 7.0. Tumors were primarily located in the right colon (52.2%) and sigmoid colon (25.6%), with most patients having stage II (35.6%) or stage III (25.5%) disease. Elective surgeries accounted for 73% of procedures, and 80.0% had curative intent, with laparoscopic surgery performed in 66.7% of cases. Only 8.3% of those undergoing curative-intent procedures received adjuvant chemotherapy. Emergent admissions were associated with more advanced cancer stages, higher rates of palliative intent procedures (45.8% versus 10.6%, p < 0.001), and more open surgeries (75.0% versus 9.1%, p < 0.001) when compared to elective procedures. Postoperative mortality was higher in the emergent group (20.8% versus 10.6%), though there was no association between the type of admission and postoperative complications. Median overall survival for all patients was 36.7 (95% CI 28.1 to 45.3) months, with significant differences between curative-intent and palliative surgeries (median of 39.8 (95% CI 32.6 to 47.0) versus 10.6 (95% CI 0.67 to 20.5) months, p = 0.015). The elective group of patients had significantly better overall survival compared to the emergent group (median of 36.7 (95% CI 30.7 to 42.7) versus 11.9 (95% CI 6.0 to 17.8) months, p = 0.01). Among the patients who underwent curative-intent procedures, there were no significant differences in overall or disease-free survival between elective and emergent groups.

Conclusions: Despite the increased complexity of managing older patients, particularly in emergent cases, these findings emphasize the importance of elective, curative-intent surgeries to optimize overall survival. Effective treatment strategies and perioperative management tailored to this age group are essential for improving surgical outcomes and extending survival in elderly colon cancer patients.

## Introduction

Colon cancer is the fourth most common malignancy, accounting for more than one million cases and about 500,000 deaths per year worldwide [1]. The incidence of colon cancer increases with age, with approximately 40% of cases diagnosed in patients older than 75 years of age [2]. Despite this, older patients are often underrepresented in clinical trials [3], which limits evidence-based recommendations for treatment and follow-up in this population. Treatment decisions are further challenged by the fact that older patients have more comorbidities and a poorer functional status when compared to their younger counterparts [2,4-8]. As the population grows older [9], the number of older patients with colon cancer is expected to rise, posing a significant challenge for healthcare professionals, and placing strain on healthcare resources. Hence, there is an imperative need for research on the treatment of colon cancer in this population. Previous studies have focused on the surgical outcomes and survival of older patients with colon cancer [2,4-7,10-15]. However, only a few specifically studied patients over 80 years of age [7,14,15].

The main goal of this study was to characterize the population of patients aged over 80 years treated surgically for colon cancer, with a specific focus on evaluating surgical outcomes, including postoperative complications and death rates, as well as long-term survival. A secondary objective was to analyze disparities between patients admitted electively and those admitted urgently.

## Materials and methods

Study population

An observational retrospective longitudinal study was conducted in a Portuguese Hospital. The hospital records were used to identify patients aged over 80 years treated surgically for histologically confirmed invasive colon cancer. Surgical treatment was characterized by either the removal of the tumor, regardless of whether the aim was curative or palliative, or by defunctioning surgery. Both elective and emergent procedures were included in the study. Emergency surgery was defined as a procedure undertaken within 48 hours of hospital admission due to an acute medical condition. This timeframe aligns with the acute care surgery classification outlined by the World Society of Emergency Surgery [16].

Exclusion criteria were as follows: tumors other than adenocarcinoma; a previous history of colon cancer; rectal cancer; overlapping lesions of the colon and the rectum; the presence of other primary malignancies besides the colon at the time of the diagnosis; exploratory laparotomy, without colonic resection or the construction of a stoma.

Variables and outcomes measures

The study examined the following patient characteristics: age, gender, Eastern Cooperative Oncology Group (ECOG) performance status, Charlson Comorbidity Index (CCI), and American Society of Anaesthesiologists (ASA) physical status classification. The tumor-related variables analyzed included tumor location, TNM stage according to the eighth edition of the American Joint Committee on Cancer (AJCC) staging manual, number of harvested lymph nodes, presence of venous, neuronal, or lymphatic invasion, presence of tumor deposits, and histological grade. The type of admission (elective versus emergent), the surgical approach (laparoscopic versus open surgery), and the surgical procedure performed were also recorded. Postoperative mortality and morbidity were defined as any death or complication occurring within the first 30 days after surgery. Postoperative complications were graded according to the Clavien-Dindo classification. Overall survival included all causes of death from the date of first admission. Disease-free survival represented the probability of not developing a first recurrence and was calculated from the date of admission.

Statistical analysis

The chi-square test and Fisher's exact test were used to assess the association between categorical variables. Mann-Whitney U test was used for the analysis of non-normally distributed continuous variables. Overall survival and disease-free survival analysis was performed using the Kaplan-Meier method. In the overall survival analysis, survival time was calculated as the time from the date of surgery to the date of death or last follow-up; patients who were still alive at the end of the study period or lost to follow-up were censored at the time of their last known contact. In the disease-free survival analysis, survival time was calculated as the time from the date of surgery to the date of recurrence or last follow-up/death. A comparison of subgroups was performed using the log-rank test. Statistical significance was set at p < 0.05. Statistical analysis was performed using the IBM SPSS Statistics for Windows, Version 27 (Released 2020; IBM Corp., Armonk, New York, United States).

## Results

A total of 90 patients were included in the study (Table 1). Most patients (73.3%) underwent elective surgery. The median age of the participants was 84.3 (IQR 6.0) years, and 41.1% were female. Forty-three percent of the patients had an ECOG performance status equal to or greater than two, and 48.9% had an ASA score greater than two. The median CCI was 7.0 (IQR 2.0). Most of the tumors (52.2%) were located in the right colon, and accordingly, the most frequently performed surgery was right colectomy. Laparoscopic surgery was performed in 66.7% of the patients. Most patients had either stage II (35.6%) or stage III (25.5%) disease. The majority of the patients (63.3%) did not have any postoperative complications. Postoperative mortality was 13.3%. Postoperative complications are depicted in Table 2.

**Table 1:** Descriptive characteristics of 90 patients aged over 80 years treated surgically (either with curative or palliative intent) for colon cancer according to the type of admission a: Mann-Whitney U test; b: chi-square; *: two subtotal colectomies and one ileocolic bypass IQR: interquartile range; ECOG: Eastern Cooperative Oncology Group; ASA: American Society of Anaesthesiologists

Patient characteristics	Total (90)	Elective surgery (66)	Emergent surgery (24)	p	Test
Age, median (IQR)	84.3 (6.0)	84.2 (5.8)	84.8 (7.0)	0.590	a
Sex					
Male, n (%)	53 (58.9)	41 (62.1)	12 (50)	0.301	b
Female, n (%)	37 (41.1)	25 (37.9)	12 (50)		
ECOG performance status					
0, n (%)	17 (18.9)	14 (21.2)	3 (12.5)	0.427	b
1, n (%)	34 (37.8)	26 (39.4)	8 (33.3)		
≥2, n (%)	39 (43.3)	26 (39.4)	13 (54.2)		
Charlson Score Index, median (IQR)	7.0 (2.0)	7 (1.3)	7 (3.8)	0.071	a
ASA physical status classification					
1, n (%)	1 (1.1)	0 (0)	1 (4.2)	0.117	b
2, n (%)	45 (50.0)	33 (50.0)	12 (50.0)		
3, n (%)	39 (43.3)	31 (47.0)	8 (33.3)		
4, n (%)	5 (5.6)	2 (3.0)	3 (12.5)		
Anatomical location					
Splenic flexure, n (%)	6 (6.7)	3 (4.5)	3 (12.5)	0.274	b
Right colon, n (%)	47 (52.2)	38 (57.6)	9 (37.5)		
Descending colon, n (%)	7 (7.8)	4 (6.1)	3 (12.5)		
Sigmoid colon, n (%)	23 (25.6)	15 (22.7)	8 (33.3)		
Transverse colon, n (%)	7 (7.8)	6 (9.1)	1 (4.2)		
Curative-intent surgery					
Yes, n (%)	72 (80.0)	59 (89.4)	13 (54.2)	<0.001	b
No, n (%)	18 (20.0)	7 (10.6)	11 (45.8)		
Surgical procedure					
Right colectomy, n (%)	45 (50)	38 (57.6)	7 (29.2)	0.002	b
Right colectomy with ileostomy, n (%)	3 (3,3)	1 (1.5)	2 (8.3)		
Splenic flexure resection, n (%)	5 (5,6)	5 (7.6)	0 (0)		
Sigmoid colectomy, n (%)	4 (4,4)	4 (6.1)	0 (0)		
Sigmoid colectomy with end colostomy, n (%)	7 (7,8)	3 (4.5)	4 (16.7)		
Left colectomy, n (%)	8 (8,9)	7 (10.6)	1 (4.2)		
Left colectomy with end colostomy, n (%)	4 (4,4)	3 (4.5)	1 (4.2)		
Derivative colostomy, n (%)	11 (12,2)	3 (4.5)	8 (33.3)		
Other procedures*, n (%)	3 (3,3)	2 (3.0)	1 (4.2)		
Total of patients with an ostomy, n (%)	26 (28.9)	10 (15.1)	16 (66.7)	<0.001	b
Surgical approach					
Open surgery, n (%)	24 (26.7)	6 (9.1)	18 (75.0)	<0.001	b
Laparoscopic surgery, n (%)	60 (66.7)	54 (81.8)	6 (25.0)		
Conversion to open surgery, n (%)	6 (6.7)	6 (9.1)	0 (0)		
Surgery complications (Clavien Dindo Classification)					
No complications, n (%)	57 (63.3)	43 (65.2)	14 (58.3)	0.523	b
Grade I, n (%)	5 (5.6)	3 (4.5)	2 (8.3)		
Grade II, n (%)	6 (6.7)	4 (6.1)	2 (8.3)		
Grade IIIa, n (%)	1 (1.1)	1 (1.5)	0 (0)		
Grade IIIb, n (%)	5 (5.6)	4 (6.1)	1 (4.2)		
Grade IVb, n (%)	4 (4.4)	4 (6.1)	0 (0)		
Grade V, n (%)	12 (13.3)	7 (10.6)	5 (20.8)		
Prognostic stage group					
Stage I, n (%)	17 (18.9)	13 (19.7)	4 (16.7)	0.04	b
Stage II, n (%)	32 (35.6)	29 (44.0)	3 (12.5)		
Stage III, n (%)	23 (25.5)	17 (25.7)	6 (25.0)		
Stage IV, n (%)	12 (13.3)	6 (9.1)	6 (25.0)		
Not determined, n (%)	6 (6.7)	1 (1.5)	5 (20.8)		

**Table 2:** Postoperative complications of 90 patients aged over 80 years treated surgically for colon cancer according to the Clavien-Dindo classification

Postoperative complications	Total (90)	Elective (66)	Emergent (24)
Grade I			
Acute kidney injury, n (%)	1 (1.1)	0 (0)	1 (4.2)
Hypokalemia, n (%)	1 (1.1)	1 (1.5)	0 (0)
Postoperative ileus, n (%)	2 (2.2)	1 (1.5)	1 (4.2)
Lower gastrointestinal bleeding, n (%)	1 (1.1)	1 (1.5)	0 (0)
Grade II			
Superficial incisional surgical site infection, n (%)	2 (2.2)	1 (1.5)	1 (4.2)
Intra-abdominal infection, n (%)	1 (1.1)	1 (1.5)	0 (0)
Urinary tract infection, n (%)	1 (1.1)	0 (0)	1 (4.2)
Lower gastrointestinal bleeding, n (%)	1 (1.1)	1 (1.5)	0 (0)
Anemia, n (%)	1 (1.1)	1 (1.5)	0 (0)
Grade IIIa			
Intra-abdominal infection, n (%)	1 (1.1)	1 (1.5)	0 (0)
Grade IIIb			
Anastomosis dehiscence, n (%)	3 (3.3)	2 (3.0)	1 (4.2)
Stoma necrosis, n (%)	1 (1.1)	1 (1.5)	0 (0)
Abdominal fascia dehiscence, n (%)	1 (1.1)	1 (1.5)	0 (0)
Grade IVb			
Small bowel perforation, n (%)	2 (2.2)	2 (3.0)	0 (0)
Anastomosis dehiscence, n (%)	1 (1.1)	1 (1.5)	0 (0)
Intra-abdominal infection, n (%)	1 (1.1)	1 (1.5)	0 (0)
Grade V			
Small bowel perforation, n (%)	1 (1.1)	1 (1.5)	0 (0)
Anastomosis dehiscence, n (%)	6 (6.7)	5 (7.6)	1 (4.2)
Respiratory infection, n (%)	4 (4.4)	0 (0)	4 (16.7)
Decompensated heart failure, n (%)	1 (1.1)	1 (1.5)	0 (0)

Eighty percent of the patients underwent curative-intent surgery (Table 3). Of these, most had either stage II (43.1%) or stage III (31.9%) disease. Peritoneal carcinomatosis was detected intraoperatively in one patient initially proposed for curative surgery. There was one case of macroscopic incomplete resection. The median number of harvested lymph nodes was 21 (IQR 17.5). Only 8.3% of patients undergoing curative-intent surgery received adjuvant chemotherapy.

**Table 3:** Descriptive characteristics of 72 patients aged over 80 years who underwent curative-intent surgery for colon cancer according to the type of admission a: Mann-Whitney U test; b: chi-square; c: Fisher exact test; d: Mann-Whitney U test IQR: interquartile range; ChT: chemotherapy

Patient characteristics	Total (72)	Elective surgery (59)	Emergent surgery (13)	p	Test
pT stage					
T1, n (%)	5 (6.9)	4 (6.8)	1 (7.7)	0.396	b
T2, n (%)	14 (19.4)	11 (18.6)	3 (23.1)		
T3, n (%)	46 (63.9)	37 (62.7)	9 (69.2)		
T4, n (%)	7 (9.8)	7 (11.9)	0 (0)		
pN stage					
N0, n (%)	50 (69.4)	42 (71.2)	8 (61.5)	0.612	b
N1, n (%)	21 (29.2)	16 (27.1)	5 (38.5)		
N2, n (%)	1 (1.4)	1 (1.7)	0 (0)		
Prognostic stage group					
Stage I, n (%)	17 (23.6)	13 (22)	4 (30.8)	0.343	b
Stage II, n (%)	31 (43.1)	28 (47.5)	3 (23.1)		
Stage III, n (%)	23 (31.9)	17 (28.8)	6 (46.2)		
Stage IV, n (%)	1 (1.4)	1 (1.7)	0 (0)		
Vascular invasion, n (%)	8 (11.1)	6 (10.2)	2 (15.4)	0.629	b
Perineural invasion, n (%)	5 (6.9)	5 (8.5)	0 (0)	0.577	c
Lymphatic invasion, n (%)	17 (23.6)	16 (27.1)	1 (7.7)	0.170	b
Tumor deposits, n (%)	3 (4.2)	2 (3.4)	1 (7.7)	0.455	b
Resection					
R0, n (%)	71 (98.6)	58 (98.3)	13 (100)	1.00	c
R1, n (%)	0 (0)	0 (0)	0 (0)		
R2, n (%)	1 (1.4)	1 (1.7)	0 (0)		
Histological grade					
G1, n (%)	33 (45.8)	27 (45.8)	6 (46.2)	0.996	b
G2, n (%)	33 (45.8)	27 (45.8)	6 (46.2)		
G3, n (%)	6 (8.3)	5 (8.5)	1 (7.7)		
Number of lymph nodes sampled, median (IQR)	21 (17.5)	23 (18)	16 (11)	0.330	
Number of lymph nodes sampled					
<12	8 (11.1)	8 (13.6)	0 (0)	0.336	c
≥12	64 (88.9)	51 (86.4)	13 (100)		
Adjuvant ChT, n (%)	6 (8.3)	6 (10.2)	0 (0)	0.583	c

When comparing patients undergoing elective surgery with those undergoing emergent surgery, we observed no association between the type of admission and gender, age distribution, ECOG performance status, CCI, ASA score, or the anatomical location of the tumor. In the emergent group, there was a higher number of patients receiving palliative surgery (45.8% versus 10.6% in the elective group), and a statistically significant association was found between the type of admission and the surgical intent (p < 0.001). In addition, a statistically significant association was observed between the type of admission and both the surgical approach (p < 0.001) and the construction of a stoma (p < 0.001), with the emergent group exhibiting a higher frequency of open surgeries (75.0% versus 9.1% in the elective group) and a greater number of patients with a stoma (66.7% versus 15.1% in the elective group). Moreover, there was a statistically significant association between the type of admission and the prognostic stage group (p = 0.04), with the emergent group having a higher number of stage III and stage IV cancers. There was also a statistically significant association between the type of admission and the surgical procedure performed (p = 0.002). In the elective group, the most frequent procedure performed was right colectomy; whereas in the emergent group, the predominant surgery conducted was derivative colostomy. Seventy-five percent of the emergent procedures were due to colonic obstruction. Although we found no association between the type of admission and the presence of postoperative complications, postoperative mortality was twice as high in the emergent group. Of note is the fact that respiratory infections were the primary cause of postoperative fatalities in the emergent group, whereas anastomosis dehiscence emerged as the most prevalent cause of mortality in the elective group (Table 2).

When comparing the emergent and elective groups considering only the patients that underwent a curative-intent surgery (Table 3), there was no association between the type of procedure performed and the pT stage, pN stage, pTNM stage, completeness of resection, histological grade, or the number of harvested lymph nodes. Even though there were no patients undergoing adjuvant chemotherapy in the emergent group, there was no statistically significant association between the type of procedure and the probability of undergoing adjuvant treatment.

The median follow-up time for all patients was 13.5 (IQR 20.2) months. The elective group had a median follow-up time of 17.9 (IQR 20.1) months, while the emergent group had a median follow-up time of 9.3 (IQR 12.9) months.

**Figure 1:**
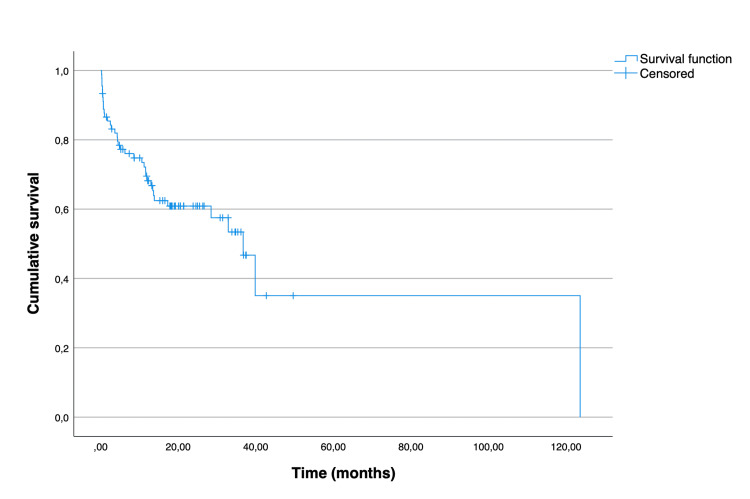
Overall survival of 90 patients aged over 80 years treated surgically for colon cancer (either with curative or palliative intent).

**Figure 2:**
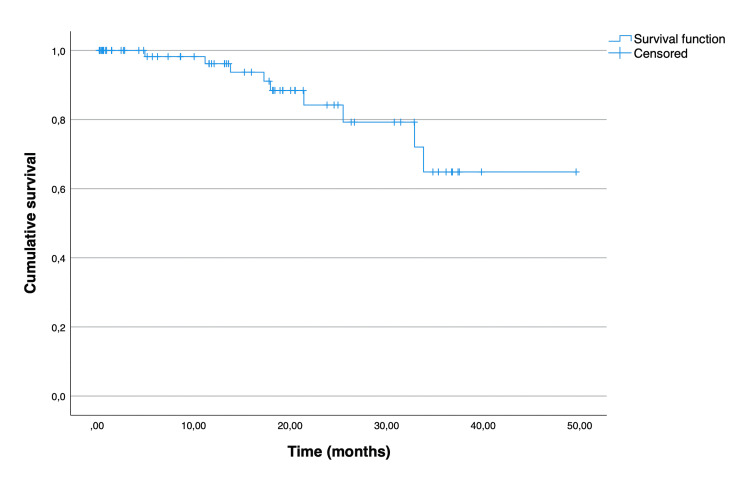
Disease-free survival of 72 patients aged over 80 years who underwent curative-intent surgery for colon cancer.

**Figure 3:**
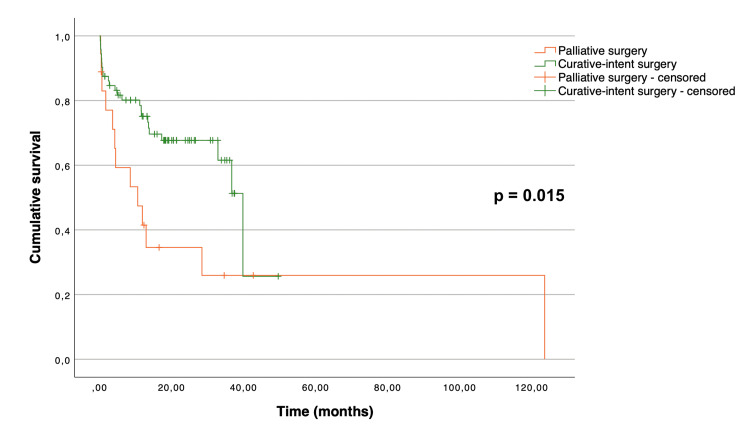
Overall survival of 90 patients aged over 80 years treated surgically for colon cancer, according to the intent of the surgery (palliative versus curative).

**Figure 4:**
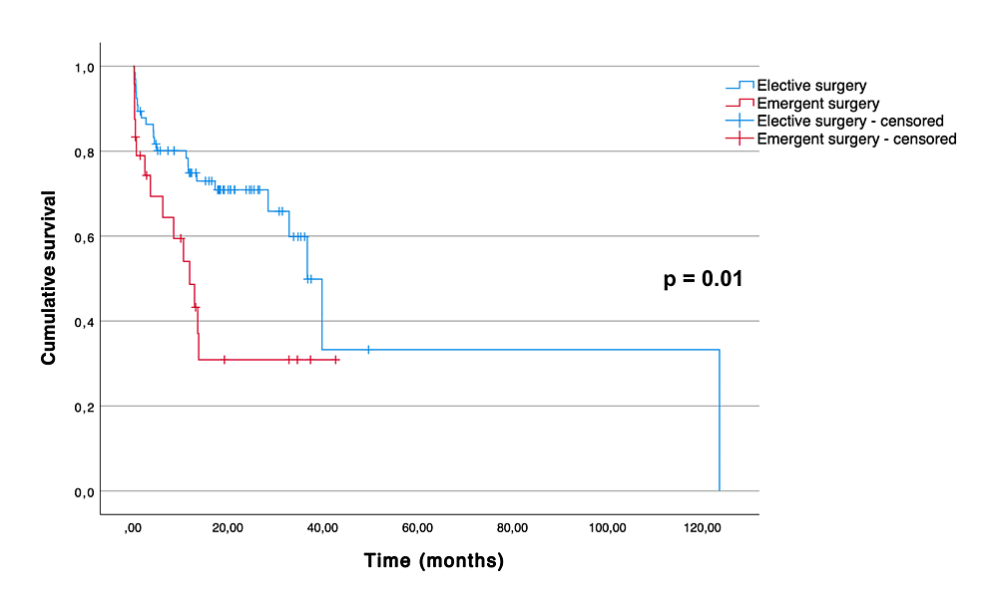
Overall survival of 90 patients aged over 80 years treated surgically (either with curative or palliative intent) for colon cancer, according to the type of admission (emergent versus elective).

**Figure 5:**
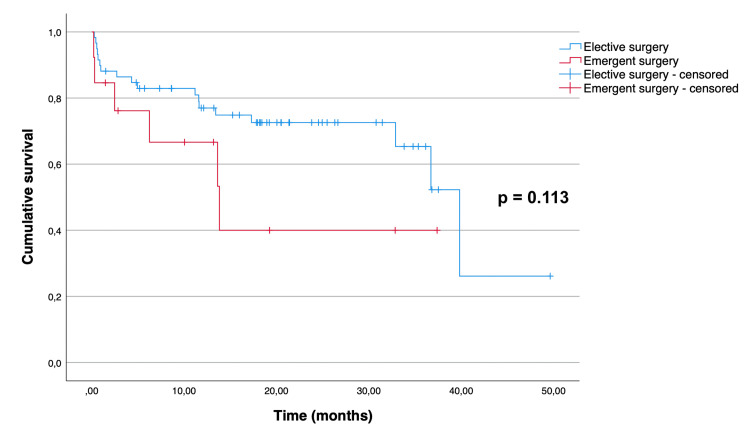
Overall survival of 72 patients aged over 80 years who underwent curative-intent surgery for colon cancer, according to the type of admission (emergent versus elective).

**Figure 6:**
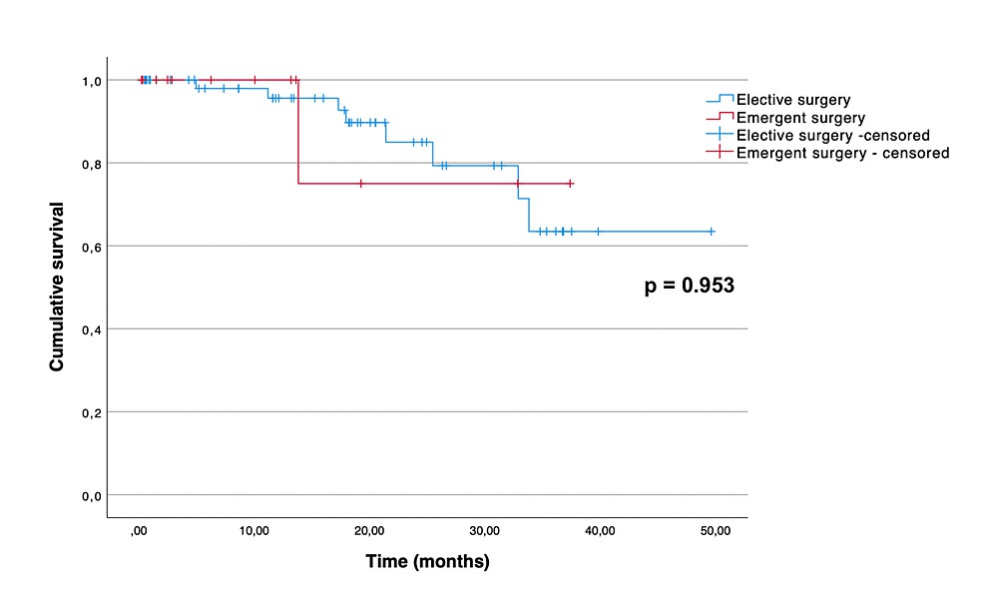
Disease-free survival of 72 patients aged over 80 who underwent curative-intent surgery for colon cancer, according to the type of admission (emergent versus elective).

The Kaplan-Meier curves illustrating overall and disease-free survival are depicted in Figure 1 and Figure 2, respectively. The median overall survival for all patients was 36.7 (95% CI 28.1 to 45.3) months. The mean disease-free survival of the patients who underwent curative-intent surgery was 40.7 months (95% CI 35.7 to 45.7). As expected, there was a significant difference between the overall survival distributions of the patients who underwent curative-intent surgery when compared to the ones who underwent palliative surgery (p = 0.015) (Figure 3). The median overall survival for the curative-intent group was 39.8 (95% CI 32.6 to 47.0) months, while in the palliative group was 10.6 (95% CI 0.67 to 20.5) months. Moreover, we found a statistically significant difference between the overall survival distributions of the emergent and elective groups (p = 0.01), favoring the elective group (Figure 4). In the emergent group, patients had a median survival of 11.9 (95% CI 6.0 to 17.8) months, while those in the elective group had a median survival of 36.7 (95% CI 30.7 to 42.7) months. Despite this difference, when considering only the patients who underwent curative-intent procedures, there were no differences in overall (p = 0.113) or disease-free survival (p = 0.953) between those groups (Figures 5, 6).

## Discussion

Studying colon cancer in older adults is of paramount importance not only due to its higher incidence in this population [2], but also to the unique challenges that these patients present in relation to treatment. In this study, we retrospectively analyzed the clinical-pathological features, surgical outcomes, and survival of 90 patients aged over 80 years who underwent surgery, either for curative or palliative purposes, for colon cancer.

There is no consensus on the precise age at which an individual is considered elderly, likely due to the realization that chronological age alone may not fully capture the diversity of health status, functional ability, and social circumstances among older individuals [17]. Probably one of the most used definitions of elderly is the one by the World Health Organization (WHO), which considers individuals aged 65 years and older as “older adults” or “seniors” [18]. Both the WHO and the American Geriatric Society define a subset of individuals known as "the oldest-old," as those aged over 80 years [19]. In our study, we used this cutoff as an inclusion criterion.

Although colon cancer is more prevalent among men [1], as individuals age, the incidence of colon cancer among women tends to increase [10,14,20,21], sometimes surpassing that of men in some studies [7,13-15,22]. However, we did not observe this trend in our population, as the majority of our patients were male.

Patients had a median CCI of 7 (IQR 2), and most had an ASA score of 2 or higher. This reflects the reality of the oldest old included, a population with a significant burden of comorbidities and reduced autonomy. This is consistent with prior research demonstrating that older individuals tend to have more comorbidities and poorer functional status compared to their younger counterparts [4-6,8,20].

Most of the patients in this study presented with right-sided colon cancer, which is in agreement with previous publications describing that the incidence of right-sided tumors increases with age reaching about 50% of cases at the age of 80 years [8,10,14,21,23]. Previous studies have shown that, although older patients typically present with a more advanced pT stage of colon cancer, they demonstrate a lower propensity for lymph node and liver metastasis compared to younger patients [21]. Accordingly, when considering all patients enrolled in the study, it was found that the majority had stage II cancer. Furthermore, among those undergoing curative-intent surgery, most presented with pT3 disease and showed no evidence of lymph node metastasis.

The role of laparoscopic surgery in colon cancer is well established, as the laparoscopic approach is associated with lower postoperative morbidity, faster recovery, and shorter hospital stays [24-30], without compromising long-term oncological outcomes [31-35]. While there is limited literature dedicated to examining the role of laparoscopic surgery in older patients, emerging evidence suggests that laparoscopy offers similar benefits in this population [11,12,36], and a randomized controlled trial has already been initiated [37]. Most of the colon cancer patients electively admitted to our institution undergo laparoscopic surgery, and the older adults are no exception. Considering that laparoscopy aims to minimize surgical trauma and physiological disruption, these benefits in postoperative recovery and reduced hospital stays are particularly important for maintaining functionality in older patients with limited reserves compared to younger individuals.

Among older adults, colon cancer surgery is associated with higher rates of postoperative morbidity and mortality when compared to younger patients. Reported morbidity rates range from 32% to 58% [4-6], while mortality rates range from 8% to 13% [4-6,14]. Some studies do not specify whether they considered only elective procedures when calculating morbidity and mortality. Nonetheless, emergent surgeries seem to be associated with a higher rate of postoperative complications than elective procedures [4,6]. The morbidity and mortality rates in our study are in line with the ones previously reported in the literature.

Lymph node yield is not only essential for accurate staging but also influences prognosis and reflects the quality of the surgery [38-40]. Prior studies have demonstrated that older patients submitted to colon cancer surgery have a lower number of harvested lymph nodes than younger patients [40-42]. It is not clear if this represents less extensive lymphadenectomies or a reduced immune response in older patients. In our study, most patients undergoing curative-intent surgery had 12 or more lymph nodes in the surgical specimen.

A disparity also exists in the administration of chemotherapy between older patients and their younger counterparts. Although most evidence suggests that adjuvant chemotherapy is similarly effective and safe for older patients as it is for younger patients, older patients are less likely to receive this treatment [5,8,20,22,43]. Patient age alone, comorbidities, and perceived minimal benefit of adjuvant chemotherapy in this population are some of the reasons for withholding chemotherapy [8]. In our study, only 8% of the patients submitted to curative-intent procedures received adjuvant chemotherapy. The administration of adjuvant chemotherapy to older patients with colon cancer in Europe varies significantly, with rates of adjuvant treatment ranging from 0.8% to 4.8% for stage II and from 3.9% to 24.8% for stage III cancer [15].

When comparing patients undergoing elective surgery with the ones undergoing emergent surgery, we found a significant association between the type of admission and staging, with the emergent group presenting a higher proportion of patients in stages III and IV. There was a significant association between the type of admission and the intent of the surgery, with a higher proportion of patients submitted to palliative surgery in the emergent group. Even though most of the tumors in the emergent group were in the right colon, similar to the elective group, the primary procedure performed in this group was not right colectomy but rather derivative colostomy. Indeed, the percentage of patients with an ostomy in the emergent group was higher than in the elective group, and there was a significant association between the type of admission and the presence of a stoma. The high proportion of patients with an ostomy may be attributed to several factors: i) the advanced disease stage, necessitating a diversionary procedure as the sole viable option for patients experiencing colonic obstruction; ii) compromised local and/or systemic conditions during surgery, rendering an anastomosis too risky; iii) the fact that emergent procedures are not exclusively performed by a team specifically dedicated to colorectal surgery. There was also a significant association between the type of admission and surgical approach, with a higher percentage of patients undergoing open surgery in the emergent group. Locally advanced tumors and the intestinal distension associated with colonic obstruction pose challenges for the laparoscopic approach, potentially accounting for the observed differences between the groups. Although an association was not found between the type of admission and postoperative complications, the mortality rate among emergent patients was roughly twice as high as that among patients undergoing elective surgery. Additionally, most postoperative deaths among patients undergoing emergent surgery were due to medical complications, contrasting with patients undergoing elective surgery, where surgical complications were the primary cause of mortality. These discrepancies in the rate and causes of postoperative mortality might be attributed to the absence of preoperative preparation in these patients, the more advanced stage of disease at presentation, and the underlying causes necessitating emergent surgery, with most of our patients undergoing emergent interventions due to obstruction of the colon. In all patients undergoing curative-intent surgery in the emergent setting, there were a minimum of 12 lymph nodes present in the surgical specimen. Data regarding the influence of the priority of the surgery on the lymph node yield are conflicting, with certain studies showing a decrease in harvested lymph nodes during emergent procedures [41,44], while others show no variation in the number of sampled lymph nodes based on the priority of the procedure [45-47]. We found no association between the type of admission and the number of harvested lymph nodes. In our study, none of the patients undergoing curative-intent procedures in the emergent setting received adjuvant chemotherapy. There was no association between the type of admission and the probability of receiving chemotherapy.

Prior studies have reported lower overall and disease-free survival in older patients compared to their younger counterparts [5-7,14]. Reasons that may explain the decreased survival in older patients with colon cancer include reduced life expectancy, elevated postoperative mortality and morbidity rates, and a lower probability of undergoing adjuvant chemotherapy. In our study, the median overall survival for patients undergoing curative-intent surgery was 39.8 (95% CI 32.6 to 47.0) months, which is in line with previous research [7]. As expected, overall survival was significantly higher in the group of patients undergoing curative-intent procedures when compared with the ones undergoing palliative surgery. Additionally, there was a statistically significant difference between the overall survival distributions of the emergent and elective groups, favoring the elective group. This may be explained by the higher number of patients in stages III and IV of the disease in the emergent group. In fact, when comparing the survival distributions according to the type of admission considering only the patients who underwent curative-intent procedures, we found no significant difference in overall or disease-free survival between the emergent and elective groups.

Study limitations

This study has several limitations. First, a retrospective single-center study is prone to several biases, including selection bias, as the patient population may not represent the general population, and information bias from potentially incomplete or inaccurate records. In this particular case, information bias could have led to an underestimation of patients' comorbidities. Second, the limited number of patients undergoing emergent surgery prevents an extensive comparison between this group of patients and those who underwent elective surgery. Third, we do not have specific information on how many patients in stage II had associated risk factors that indicated the need for chemotherapy, which limits our ability to draw conclusions about the proportion of patients undergoing chemotherapy. Larger multicenter studies are needed to enhance the accuracy and applicability of our findings, allowing for more definitive conclusions regarding the treatment and outcomes of patients.

## Conclusions

This study provides valuable insights into the treatment patterns and outcomes of older patients undergoing colon cancer surgery. Elective surgery was relatively safe in the oldest old. However, the contrast in outcomes between emergent and elective surgeries underscores the necessity for enhanced preoperative assessment and possibly more aggressive early interventions to reduce the need for emergent procedures. These findings advocate for a tailored approach to treatment and vigilant perioperative strategies to improve survival outcomes and quality of life for older patients with colon cancer.
